# Task Sharing and Task Shifting (TSTS) in the Management of Africans with Hypertension: A Call For Action-Possibilities and Its Challenges

**DOI:** 10.5334/gh.1301

**Published:** 2024-02-22

**Authors:** Oluseyi Ademola Adejumo, Reuben Mutagaywa, Florence Koryo Akumiah, Adeseye Abiodun Akintunde

**Affiliations:** 1 Department of Internal Medicine, University of Medical Sciences, Ondo State, Nigeria; 2 Department of Internal Medicine, Muhimbili University of Health and Allied Sciences, Tanzania; 3 Muhimbili Orthopedic Institute, Tanzania; 4 Department of Medicine and Therapeutics, Korle-Bu Teaching Hospital, Ghana; 5 National Cardiothoracic Centre, Korle Bu, Ghana; 6 Department of Medicine, Faculty of Clinical Sciences, Ladoke Akintola University of Technology and LAUTECH Teaching Hospital, Ogbomoso, Nigeria

**Keywords:** Hypertension, Task-shifting, Low and Middle Income Countries (LMIC)

## Abstract

Hypertension is a leading cause of mortality globally and one of the most common risk factors for cardiovascular disease. Diagnosis, awareness, and optimal treatment rates are suboptimal, especially in low- and middle-income countries, with attendant high health consequences and grave socioeconomic impact. There is an enormous gap between disease burden and physician-patient ratios that needs to be bridged. Task sharing and task shifting (TSTS) provide a viable temporary solution. However, sociocultural, demographic, and economic factors influence the effective uptake of such interventions. This review discusses the dynamics of TSTS in the African context looking at challenges, feasibility, and approach to adopt it in the management of hypertension in Africa.

## The Burden of Hypertension

Hypertension is a long-term condition where blood pressure is persistently elevated. It is the leading cause of death worldwide, affecting more than 1.4 billion people and accounting for more than 28,000 deaths each day [1]. Hypertension is the most common single risk factor for cardiovascular-related events and deaths worldwide. Over the past decade, Africa has been characterized as the world’s fastest-growing economy but is also in a precipitous epidemiologic health transition [2,3].

The global burden of disease attributable to hypertension has significantly increased in prevalence from about 4.5% in 2001 to 7% in 2010 and is predicted to rise to as high as 29.2% (28.8%–29.7%) by 2025 [1,2,3] and is projected to remain so by 2040 [4]. Compared with other WHO regions, Africa has the highest prevalence of hypertension with an overall prevalence of 46% in adults aged 25 years and above [5]. With this scourge of a rising prevalence of hypertension are the issues of poor awareness, low treatment and control rates of hypertension across the region with its attendant increased cardiovascular risk [6,7,8]. Hypertension, therefore, stands in this region of the world as the most common cause of >200 million disability life years per year as a result of stroke, coronary artery disease, peripheral arterial disease, congestive heart failure (HF), and chronic kidney disease, and poses additional challenges on the longstanding burden associated with communicable diseases and the ongoing HIV/AIDS pandemic [9].

## The Peculiarities of Africans and Hypertension

Sub-Saharan African (SSA) countries are experiencing one of the most rapid epidemiological transitions, but with a very challenging health system to tackle the menace [10,11,12]. SSA is faced with two epidemics: a menace of communicable diseases such as malaria, tuberculosis, and HIV and a rapidly rising burden of non-communicable diseases including hypertension, stroke, kidney disease, and diabetes mellitus. With limited resources, most countries are barely able to cope with the required health investments/budgets to tackle the double-edged sword [10]. The consequence of an increasing burden of hypertension in SSA is therefore likely going to lead to grave consequences resulting in a poorer proportion of people with adequate blood pressure control and diagnosis which in turn will lead to high morbidity and mortality from potentially preventable complications such as renal failure, stroke, heart failure, diabetes, and peripheral arterial disease [13,14]. Ethnic differences in hypertension pathophysiology, incidence and prevalence, treatment, control rate, and outcomes have been established in the literature [15]. There is a higher prevalence in blacks compared to whites, with an associated younger age at incidence, faster progression from prehypertension to hypertension, and more severe hypertension [16]. Multiple and varied factors have been postulated to contribute to these differences, including genetics, psycho-socio-economic factors, differences in renal and endocrine physiology, differential susceptibility of target organs, et cetera. These may contribute to the poorer outcomes observed in blacks, including the excess mortality and morbidity from hypertension-related diseases out of proportion to the prevalence of hypertension itself [16].

The detection rates, treatment, and control rates in women have been reported to be better than in men in both SSA and high-income countries [14,15,16]. The seemingly higher detection rate among women may be related to increased chances of having blood pressure measured on contact with a health facility during pregnancy and related health conditions. Women are also likely to probably accept more readily the diagnosis of hypertension even in the absence of symptoms and recognize the need to stay healthy to support their families and are more willing to comply with treatment and get controlled. The generally low levels of detection, treatment, and control of hypertension reported emphasize the difficulty in managing chronic health conditions that are usually not associated with symptoms and yet need lifelong management to ensure control. Health spending is generally limited in SSA where the majority of people have to pay out of pocket for health care utilization and treatment. Therefore, it is almost predictable that health conditions like hypertension that require lifelong follow-up, treatment, and control have received little or no attention from governments across the African continent [17,18,19,20,21]. Moreover, many people in Africa have limited access to non-communicable disease (NCD) care, especially in primary care settings, which is the first point of contact for people residing in rural areas. Factors contributing to this include poor population awareness/knowledge of NCDs, long distances to health facilities, and prevalent use of herbal and traditional medicines [22]. Also, failure to adhere to life style modification and a lack of medications such as that was reported from Malawi is a common finding in which first-line antihypertensive medications are mostly unavailable in health centers and inconsistently available in hospitals at the district level [23].

A few programs have been established in SSA over the years for the effective control of hypertension such as the African Control of Hypertension through Innovative Epidemiology and a Vibrant Ecosystem (ACHIEVE): novel strategies for accelerating hypertension control in Africa [17]. An adjusted correlated random effects model in Africa revealed that systolic blood pressure was significantly higher among respondents from households that reported the death of a household member and a reduction in grant income and remittances. They also found no significant association between systolic blood pressure and neighborhood income level. In a country with social and economic challenges, the results indicate that grief and negative financial events are adversely associated with blood pressure, which may explain in part the significant burden of hypertension in low- and middle-income countries [24].

## Health-Worker Distribution in African Countries

Sub-Saharan African populations and economies are growing at unprecedented speed with a population growth of 2.7% annually and economic growth of 4.4% annually [25]. Despite having the world’s largest disease burden, Africa has the lowest ratio of health workers per population [26,27]. At the same time, all African countries have committed to providing universal health care (UHC) to their populations, which will require a dramatic expansion in the provision of health services. Yet a number of bottlenecks are making this difficult to achieve [26]. For example, in Tanzania, a bill for UHC has just been passed recently [28]. The precarious situation of health workers in Africa was highlighted in a recent work by IntraHealth International conducted with the World Health Organization, the estimated global shortage of health workers, which is the largest in Africa, will expand from 12 million to 18 million by 2030, with a 6 million shortage in Africa [27]. With the fastest-growing population in the world and some of the worst high school graduation rates, Africa will be challenged to train enough health workers to meet needs [27].

## Task Shifting and Task Sharing (TSTS)

Task shifting occurs when a task is transferred or delegated while task sharing occurs when tasks are completed collaboratively between providers with different levels of training [29]. Task shifting and task sharing (TSTS) involves the redistribution or delegation of health care tasks within the workforce and communities in such a way that the quality of care is not affected. In this case, the targeted non-physician health workers (NPHWs) will undergo some specific training before they take up assigned tasks which are usually different from their routine tasks. The primary aim of TSTS strategy is to effectively utilize existing human resources for health in order to deliver quality health services to the population without compromising standards of care. The TSTS strategy has been widely and successfully utilized in maternal health care services and communicable diseases especially in the care of tuberculosis and HIV patients [29,30,31,32]. TSTS is, however, also gaining ground in management of non-communicable diseases such as hypertension, heart failure, diabetes mellitus, and cardiovascular diseases with positive outcomes [33,34,35,36,37,38,39].

Despite the fact that TSTS has the potential to solve the shortage of human resources for health, it should only be used as a temporary and not a permanent measure to solve the issue of shortage of human resources in health care systems. There must be genuine and consistent efforts in implementing policies that will ensure that there are adequate numbers of health care practitioners (HCPs) to deliver quality health care to the populace. Expert care and supervision should not be compromised or undermined by strategies meant to compensate for a temporary crisis.

### Task Shifting and Sharing in Low- and Middle-Income Countries

TSTS has been implemented in the management of non-communicable diseases, especially hypertension in low- and middle-income countries (LMICs), particularly in different parts of Africa with some positive outcomes [33,34,38]. The NPHWs who commonly take up TSTS roles are allied health care workers like nurses, community health nurses, community health extension workers (CHEWs), radiographers/radiologists, and pharmacy technologists/pharmacists [39]. In a scoping review of studies on task-shifting roles and intervention on some cardiovascular diseases involving 10 African countries, Okpechi et al. [40] reported that hypertension management tasks were more shifted to nurses compared to other NPHWs such as pharmacists and CHEWs. They also reported that blood pressure control was significantly better in nurse-led hypertension management compared to other NPHWs. The aspects of hypertension management commonly shifted to NPHWs were hypertension screening and detection, education and counseling, treatment, and adherence follow-up of clients with hypertension. In Cameroon, Kengne et al. [41] reported a significant reduction in blood pressure values in nurse-led management of hypertension when the patients were followed up for a period of 26 months. A comparable level of effectiveness was reported in a nurse-led hypertension management in primary health facilities compared to a physician-led hypertension management in secondary level of care in Democratic Republic of Congo [42]. Nurses have been shown to adhere well to protocol-guided management of non-communicable diseases such as hypertension, diabetes mellitus, asthma, epilepsy, and sickle cell when these tasks are shifted to them [37]. The patients’ outcomes in the above studies clearly provide a potentially viable option to provide quality health care to the population despite shortage of physicians in most of the LMICs.

Task-shifting interventions aimed at reduction of cardiovascular risk in LMICs have equally been found to be effective [36]. A systematic review and meta-analysis of three clinical randomized control trials (RCTs) on the effect of task-shifting strategies involving NPHWs on cardiovascular risk reduction showed a significant reduction in blood pressure and glycated hemoglobin in patients with hypertension and diabetes mellitus, respectively [36]. The adopted strategies in the study included regular education and training; supervision and feedback from higher cadre health professionals; provision of follow-up home care services for continuity of care; and use of treatment algorithms. Similarly, this decentralized strategy of NCD care led by non-physician providers made specialized cardiac care available in the rural population in countries like Malawi [39]. This scenario is important because about 63% of Africa remains rural and the poorer Africans are likely to reside in rural areas [43]. The strategy was associated with a high rate of retention in clinical follow-up and in significant symptomatology improvement. The lessons learned from Malawi could be disseminated to other sub-Saharan African countries.

A systematic review and meta-analysis of 63 studies on the effectiveness of task shifting in hypertension management in LMICs showed a significant reduction in both systolic and diastolic blood pressure [33]. The study also showed better blood pressure control in patients managed by NPHWs with higher level of training such as nurses and pharmacists compared to others like dieticians and CHEWs. The activities carried out by these NPHWs include health education on lifestyle, protocol-based management, screening, stratification, triage, monitoring, and follow-up for medication adherence. Another systematic review and meta-analysis on the effect of task-shifting intervention in the management of patients with diabetes mellitus showed a moderate reduction in fasting blood glucose and glycated hemoglobin when NPHWs such as dieticians, pharmacists, community health workers, and nurses were actively involved [35]. This study also showed that glycemic control was better in interventions led by pharmacists compared to other NPHWs. The findings of these studies also emphasized the importance of requisite knowledge for the successful implementation of the TSTS policy.

In some community health centers in Ghana, hypertensive patients with uncontrolled blood pressure had significantly better reduction in systolic and diastolic blood pressure when nurse-led TSTS strategy was combined with provision of health insurance compared with those who only had health insurance coverage [44]. The TSTS strategy used by the trained community nurses included the WHO clinical decision support for the management of cardiovascular disease via easy-to-follow algorithms, lifestyle counseling, and drug treatment protocols. The nurses were able to use treatment algorithms to initiate antihypertensive medications and modify treatment [38]. This report also highlights the importance of allowing qualified NPHWs who have been adequately trained to initiate and prescribe antihypertensive treatment using treatment protocols in the successful implementation of TSTS policies.

In China, the report of a RCT by Miao et al. [45] showed that blood pressure control, patients’ satisfaction, and self-care behavior among those with uncontrolled hypertension were significantly better in nurse-led management using protocol compared to the conventional physician-led clinic management. The protocol involved regular home visits, regular phone follow-ups, counseling on lifestyle modifications such as an increase in physical activity, smoking cessation, restriction on alcohol consumption, and medication adherence. Another RCT by Goudge et al. [46] done in the rural part of South Africa showed that hypertension clinics where lay health workers worked with nurses had better and improved clinic functioning and overall attendance compared to clinics without lay health workers. This study showed that the efforts of lay health workers could be complementary to the roles of nurses towards achieving better blood pressure control in those with hypertension. In Argentina, community health worker-led home intervention such regular health education, home blood pressure monitoring, blood pressure audit, provision of feedback and regular communication with patients via text messages in addition to physician intervention achieved significantly better blood pressure control among low-income patients with hypertension when compared with physician-led intervention alone [47].

There are also studies that have reported the effectiveness of using TSST strategies to incorporate hypertension care in the management of those living with HIV [48]. People living with HIV now have better survival rates with the advent of HAARTs which has revolutionized and significantly improved the management of HIV. However, the incidence of non-communicable diseases such as hypertension has been reported to be high in HIV patients [49]. A pragmatic approach to effectively manage this coexisting condition is by incorporating hypertension care into the HIV treatment program, which is principally driven by NPHWs, especially nurses [48,50].

### Barriers to Successful Implementation of TSTS Policy and Potential Solutions

Despite the prospects of TSTS strategies in addressing the deficiencies created by inadequate human resources for health in the delivery of quality health services to the population in LMICs, there are some identified barriers to the successful implementation of the TSTS strategies. Learning from previous experiences and addressing the encountered challenges will be pivotal to initiating a seamless and successful implementation of the TSTS strategies in hypertension management. Some of the identified barriers are discussed below.

#### Inadequate training and supportive supervision

TSTS usually involves the assignment of new roles and responsibilities to lower cadre health workers. These roles and responsibilities are above the usual scope of their training and the NPHWs may not have the requisite knowledge and capacity to discharge such assigned roles. This naturally comes with apprehension among those taking up these tasks and also raises concerns of inadequate quality care amongst the clients that would receive treatment. This therefore necessitates the need for regular and adequate training coupled with supportive supervision of NPHWs to ensure a successful and smooth TSTS. This will equally address the concerns of those to be managed.

Previous studies have emphasized the pivotal role of training and supportive supervision for NPHWs in successful implementation of TSTS strategies [51,52,53]. A study conducted in Nigeria by Aifah et al. [54] reported a major recommendation by nurses which reiterates the need for adequate training and supportive supervision for them to successfully incorporate TSTS in hypertension management of HIV patients. Gyamfi et al. [52] also reported that community health nurses were more effective in their interpersonal skills and various aspects of hypertension management following training. There are also other studies that reported significant improvement in knowledge of hypertension management by NPHWs following training [53,54]. Training will also address the concerns amongst stakeholders that border on the fact that TSTS strategies could affect patients’ safety and compromise the delivery of quality health care to the populace when the role of high cadre health workers such as physicians is shifted or shared with lower cadre health workers or non-health workers.

#### Negative perception of TSTS by higher cadre health workers, physicians and policy makers

Higher cadre health workers such as physicians, pharmacists, and medical laboratory scientists usually have their professional roles and responsibility shifted or shared with lower cadre health workers and lay workers depending on the situation in order to meet the health demands of the people. The perception of these higher cadre health workers is important for the successful implementation of this policy.

The report by Mijovic et al. [51] suggests that health workers whose roles and responsibilities are being shifted or shared may be reluctant to do so for fear that their influence in the health care sector could diminish and therefore making them irrelevant with time. This will likely lead to difficulty in getting their cooperation if their perceived insecurity and other concerns are not adequately addressed. This can, however, be addressed by ensuring that all important stakeholders including the general public are carried along in the process of developing the TSTS framework and its implementation. There should widespread consultation and open discussion so that grey areas, concerns, and negative perceptions can be analyzed and resolved. A qualitative study in Ghana assessed stakeholder perceptions and emphasized the importance of understanding stakeholder’s perceptions of evidence-based task-shifting interventions as integral to the uptake and sustainability of these interventions into ‘real world’ settings [55]. Physicians whose tasks are being shifted may also be apprehensive of redundancy and the quality of service delivery by the NPHWs. In Uganda, decision and policy makers found the concept of task shifting unacceptable due to the perceived incompetence of NPHWs citing cases of failed minor surgery, inappropriate medicine use, overwork, and inadequate support supervision [56].

Stakeholder engagement must be prioritized in the development of the framework and implementation plan for TSTS interventions. All the various health worker’s associations should also be part of the process of formulation and implementation of the TSTS policy. The importance of adequate engagement of stakeholders can be learned from the Kenyan experience where a health professional was involved in a legal tussle with the government because his professional body and the general public where not carried along during the stage of formulation of a TSTS policy [57,58]. The ministry of health of Kenya was restrained from implementing the TSTS policy in some aspects of the health system following a court judgement that was in favor of the petitioner [59]. This Kenyan experienced clearly underscores the importance of stakeholder involvement and consultation in the successful implementation of TSTS policy and strategies.

#### Absence of regulatory framework

TSTS has not been well supported by appropriate regularly framework and policies [57,60,61]. This constitutes a major barrier to the successful implementation of TSTS strategies. Roles and job descriptions should be well defined and backed up by appropriate regulatory framework in order to ensure quality of care and patients’ safety. Development of supportive framework may require amendment of existing laws regulating the activities of some health care practitioners. Any such amendment will require extensive consultations with major stakeholders such as the Ministry of Health, professional licensing bodies, professional associations, and the legislature of the concerned countries. For example, one of the major challenges faced by some NPHWs such as CHEWs in discharging their duties under TSTS arrangements is their inability to prescribe medications. This is because some countries do not have regulatory policies which enable them to do so. Implementation of policies to empower such NPHWs to be able to prescribe antihypertensive medications will contribute significantly to the success TSTS. This position is supported by the report of Ogedegbe et al. [44], which showed better blood pressure control in clients with uncontrolled hypertension in cases where trained community nurses were enabled to initiate, prescribe, and modify treatment using treatment algorithms.

#### Career path, compensation/financial constraints and reward

TSTS creates additional responsibility for lower cadre of health workers that would be taking up additional tasks. There is a tendency to overwork them, and this may consequently reduce their overall productivity, especially when they are not sufficiently motivated or compensated. Despite the additional responsibilities they bear, new career titles are not usually assigned [62]. There must be a clear path for career progression and a reward system for the lower cadre staff who will be taking on additional tasks. This is necessary to ensure successful implementation of TSTS policy. Lambrou et al. [62] reported that both financial and nonfinancial rewards led to better job satisfaction and motivation among both medical and nursing personnel.

## Lessons from the African School of Hypertension

The maiden edition of the African School of Hypertension (ASH) took place over a five-month period in 2022 [63]. One of the main objectives of the ASH was to provide training and supportive supervision for NPHWs across the African continent. The school provided training for 94 NPHWs across eight African countries (Cameroon, Gambia, Ghana, Kenya, Malawi, Nigeria, Sudan, and Tanzania) on the prevention, diagnosis, investigation, and treatment of uncomplicated hypertension. The training was facilitated by hypertension experts from different parts of Africa. The training was in two phases: namely, the interactive lecture phase where virtual lectures were delivered by facilitators for a period of 8 weeks and the mentoring phase for a period of 12 weeks. The mentoring phase was facilitated by Oyoyo wellness app, which was purposely designed for supportive supervision of the trainees. The trainee had field experience of screening people for hypertension, initiating treatment based on protocol in countries whey they were allowed and referral of diagnosed hypertensive patients. These field activities and experiences were closely supervised by the in-country mentors. At the end of the first session of training, 92 participants were certified and endorsed by ISH, OMRON Academy, and African School.

There are notable lessons that could be learnt on how to provide training and supportive supervision on hypertension management for NPHWs from the maiden edition of the ASH. First is the fact that the rapid decline in the number of health workers especially in sub-Saharan Africa and the potential for its further decline has made TSTS a veritable option in the management of chronic diseases like hypertension. We also demonstrated that with careful planning and engagement of stakeholders including government agencies, regulatory bodies, and the public, TSTS can be a successful attempt at managing uncomplicated hypertension as a chronic condition just like HIV and tuberculosis. Furthermore, the program demonstrates the heightened willingness and interest demonstrated by NPHWs to participate in the management of uncomplicated hypertension in SSA. We also demonstrated the feasibility of inculcating technologies that can further aid the effectiveness of the program in the management of uncomplicated hypertension using TSTS by NPHWs. Above all, this program provides a great hope for inculcating TSTS in the management of uncomplicated hypertension in SSA to combat the rising epidemic of cardiovascular disease and its complications. The ASH provides a good opportunity for NPHWs to be adequately trained and supervised on hypertension management towards a successful implementation of TSTS policies. There has been continued growing interest among the participants of the maiden edition of the ASH with many other NPHWs willing to attend future training [63].

## Conclusion

The TSTS strategies hold huge promise for improved management of non-communicable diseases especially hypertension in LMICs. The exodus of physicians from LMICs to developed countries in recent times has created a significant deficit in the personnel of health systems of most developing countries. TSTS has been shown to have some positive outcomes and the strategy will suffice to temporarily address the effect of the growing health personnel deficit in most LMICs pending a holistic resolution of the issue of physicians’ shortage. However, for TSTS to be successful, there is a need to ensure proper training and supervision of NPHWs, adequate compensation for the additional tasks and proper sensitization and consultation of all stakeholders to ensure their buy in and to also ensure that all concerns and fears are allayed and adequately addressed prior to the roll out of the policy. The main call to action is therefore that African nations will implement TSTS strategies alongside primary health care deliveries using NPHWs with organized training, retraining, and provision of incentives to ensure that uncomplicated hypertension is well managed among the population and CV risk is therefore potentially reduced significantly.

## Appendix

**Table 1:** Relevant literature reviewed for discussion.

S/N	AUTHOR	OBJECTIVE OF STUDY	EFFECTIVENESS	CHALLENGES

1	Farley et al. [30]	Cross-sectional study on effectiveness of TSTS on management of MDRTB in South Africa	This study found that the success rate achieved in the clinical nurse practitioner lead treatment of MDRTB using TSTS was significantly higher than the national average in South Africa. Nurse-led treatment did not compromise patients’ outcomes.	

2	Dawson et al. [31]	Review of studies on TSTS in maternal and reproductive health in low-income countries	TSTS improved availability and access to quality maternal and reproductive health services. Cost effectiveness	In-service training Poor staff coordination and training Lack of equipment and drugs Supervision Career progression Incentives

3	Callaghan et al. [32]	Systematic review of task shifting on HIV care in sub-Saharan Africa	Improve access to HAARTs especially in the underserved areas Cost effectiveness Improve overall quality of care	Proper definition of roles and responsibilities to be shifted Inter-professional rivalry

4	Atand et al. [33]	Systematic review and meta-analysis on effectiveness of TSTS on blood pressure control among adults in low- and middle-income countries	TSTS was effective in blood pressure control, especially when health workers who are more educated such as nurses and pharmacists were involved	Inadequate supply of medications and equipment required for hypertension management

5	Ogungbe et al. [34]	Systematic review and meta-analysis on effectiveness of hypertension management using TSTS in low-income countries	Team-based hypertension management was effective in blood pressure control. Its effectiveness was dependent on the level of training of the non-physicians	

6	Some et al. [37]	Cross-sectional study on adherence of nurses to protocol for managing non-communicable disease during TSTS program in Kenya	Nurses have the capacity to effectively participate in TSTS by following treatment protocol and guidelines.	

7	Fisher et al. [38]	Prospective cohort study on the effectiveness of a remote non-physician hypertension management program	Remote BP management without involvement of physicians was found to be feasible	Physicians’ perception of losing autonomy or client base

8	Mailosi et al. [39]	Cross-sectional study on effectiveness of heart failure management by trained non-physician providers in Malawi	Trained non-physician health workers successfully treated patients with heart failure. They also had a higher rate of retention of patients.	Slightly higher mortality amongst patients managed for heart failure

9	Opkechi et al. [40]	Review of studies on task-shifting roles, intervention and outcomes for kidney and cardiovascular health service delivery among Africans	TSTS is effective in the diagnosis, creation of awareness and management of hypertension, diabetes mellitus, and kidney diseases.	Effectiveness of non-physicians health workers such as nurses, pharmacists, community health workers in their TSTS role is limited by their level of training and expertise

10	Kegne et al. [41]	Cross-sectional study on effectiveness of nurse-led hypertension management in Cameroon	Nurse-led hypertension clinics were effective in management of hypertension in both rural and urban communities.	Blood pressure control was more difficult to achieve in patients with hypertension who have additional co-morbidities such as diabetes mellitus.

11	Lulebo et al. [42]	Cross-sectional study on effectiveness of task shifting in hypertension management in Congo	Management of hypertension can be effectively achieved in primary health centres	There is absence of guideline on global assessment of cardiovascular risk assessment at primary level of care

12	Ogedegbe et al. [44]	Randomized control trial that compared the effectiveness of blood pressure control in those on health insurance coverage alone compared to those on health insurance coverage and nurse-led TSTS in Ghana	The combination of TSTS and provision of health insurance coverage achieved better blood pressure control in patients with hypertension compared with those on health insurance coverage alone.	There was no enabling health policy to support antihypertensive prescription by non-physician health workers such as nurses in Ghana.

13	Miao et al. [45]	Randomized control trial that assessed the effectiveness of nurse-led hypertension management model in health care in a Chinese urban community	Nurse-led hypertension management led to significant improvement in blood pressure control in those with uncontrolled hypertension. In addition, TSTS was led to better clients’ satisfaction with health services and improvement in their self-care behavior	

14	Goudge et al. [46]	Randomized control trial that assessed the effect of lay health workers in supporting provision of integrated chronic care in rural in primary health care clinics in South Africa	TSTS with lay health workers did not improve blood pressure control of those with hypertension; however, there was improvement in organization of clinic and clinic attendance.	Inadequate supply of medications and functioning sphygmomanometer Lack of spacious consulting room and large patient population relative to available health workers

15	He et al. [47]	Randomized control trial that assessed the effect of community health worker–led multicomponent intervention on blood pressure control among low-income patients with hypertension in Argentina	Community health worker-led home-based multi-component intervention significantly improved blood pressure control in those with uncontrolled hypertension.	

16	Duffy et al. [48]	Review of studies on different models of non-communicable disease (NCD) and HIV integration in low-income countries	Integration of NCD and HIV services could improve quality of NCD care and treatment outcomes.	Identified challenges were NCD supply chain, human resource for health, referral systems, patient education, stigmatization, and patient records, monitoring, and evaluation.

17	Mijovic et al. [51]	Systematic review of studies on TSTS experiences of health care workers in sub-Saharan Africa	TSTS was associated with better communication among health team and rational distribution of work.	For low cadre health workers: Inadequate compensation, lack of clear career path, high work burden, compromising patients’ safety and working beyond the scope of practice For high cadre health workers: Perception of diminution of their roles and influence in health sector

18	Gyamfi et al. [52]	Cross-sectional study that assessed the effect of training of nurses on their knowledge and practice hypertension management during task shifting in Ghana	Training of nurses in various aspects of hypertension management led to improvement in their knowledge, interpersonal skills, and patient education skills.	

19	Labhardt et al. [53]	Cross-sectional study that assessed the effectiveness of task shifting to non-physicians in hypertension and diabetes mellitus management in rural health facilities in Cameroon	Training of nurses in the management of hypertension and diabetes mellitus before commencement of TSTS led to improvement in capacity and knowledge to manage these conditions. Patients with diabetes mellitus and hypertension managed by nurses had significant improvement in their blood glucose and blood pressure control.	Low case-detection rate of hypertension and diabetes mellitus High attrition rate among enrollees with hypertension and or diabetes mellitus

20	Aifah et al. [54]	Cross-sectional study that assessed the perception of nurses to incorporation of hypertension management into HIV program in Nigeria using TSTS strategies	Nurses opined that incorporation of hypertension care in the existing HIV program through TSTS was highly feasible and has potential of reducing the burden of non-communicable diseases.	Limited hypertension knowledge among nurses who have not been specially trained Inadequate non-physician health workers to participate in TSTS Supportive supervision

21	Iwelunmor et al. [55]	Cross-sectional study that assessed the perception of stakeholders to hypertension management in Nigeria using TSTS strategies	TSTS led to reduction in workload of higher cadre health workers and improved patients’ awareness of their health condition.	Inadequate training and supportive supervision Lack of supportive leadership and inadequate facilities to manage hypertension The need for regular retraining due to high turnover of already trained staff Lack of awareness of treatment protocol and referral guidelines on hypertension

22	Baine et al. [56]	Cross-sectional study that assessed the perception of policy and decision makers on TSTS in hypertension management in Uganda		Lack of national policy and guidelines on TSTS Inadequate competency of low cadre health workers Lack of support by policy and decision makers Inadequate supportive supervision Non-involvement of health professional regulatory bodies Lack of adequate training, remuneration, and recognition of less skilled health workers involved in TSTS

23	Joshi et al. [57]	Systematic review of studies on TSTS for management of NCD in low- and middle-income countries	TSTS improved health outcomes such as blood pressure control and medication adherence when compared with conventional health care. TSTS was found to be cost-effective.	Restriction of prescription of medications Inadequate supply of medicines

24	Kinuthia et al. [58]	Review of TSTS policies and guidelines in Kenya	TSTS improved health outcomes such as blood pressure control and medication adherence. It was also found to be cost effective.	Irregular supply of blood pressure medications and restriction of medication prescription Problem of retention of trained staff Absence of treatment guidelines Inadequate facilities such as glucose meters and sphygmomanometer

25	Munga et al. [60]	Cross-sectional study on TSTS experience in Tanzania	TSTS has the potential of improving access to health care in remote areas and increasing the retention of health workers in these areas as well.	Compromise of quality of care given to the patients Absence of monitoring and evaluation of the TSTS process Lack of supportive supervision

26	Ferrinho et al. [61]	Cross-sectional study that assessed experiences and opinions of health workers about TSTS in Zambia and Mozambique		Lack of career progression and recognition Lack of remuneration Increase workload for low and middle cadre health workers. Inadequate staffing and lack of facilities Lack of formal policy on TSTS
